# Investigating the Impact of Seasonal Input Stream Fluctuations on Post-Consumer High-Density Polyethylene Composition and Processing

**DOI:** 10.3390/polym17131828

**Published:** 2025-06-30

**Authors:** Pia Fischer, Elena Berg, Christian Hopmann, Rainer Dahlmann

**Affiliations:** Institute for Plastics Processing (IKV) in Industry and Craft, RWTH Aachen University, Seffenter Weg 201, 52074 Aachen, Germany

**Keywords:** batch fluctuations, film extrusion, high-density polyethylene, injection moulding, material analysis, plastics processing, post-consumer recycled material

## Abstract

The recycling of plastics collected from household waste to produce post-consumer recycled (PCR) materials is a critical step of sustainable waste management. However, the processing of PCR materials presents unique challenges, particularly in the context of seasonal input stream fluctuations and resulting PCR material composition variations. Within this paper, the influence of batch-to-batch fluctuations on the processing stability and product properties of high-density polyethylene (HDPE) PCR from the German municipal waste system is analysed. It examines how variations in batch composition affect key parameters such as processing data (injection pressure, torque), mechanical properties (tensile strength, E-modulus, impact strength), and product quality (gel formation, part dimensions, part weight). Therefore, six consecutive household HDPE PCR material batches are analysed regarding their composition, contaminations, and rheological characteristics through ashing, differential scanning calorimetry, high-temperature gel permeation chromatography, and high-pressure capillary rheometry. The batches are then processed using blown- and cast-film extrusion as well as injection moulding, and the resulting process stability and product quality are analysed. The results show a strong correlation between thermal properties, such as crystallisation enthalpy, molecular weight, polypropylene (PP) content, varying batch viscosities, and changes in processing data as well as the resulting product properties.

## 1. Introduction

The use of post-consumer recycled (PCR) plastics has gained increasing attention as industries seek to close the loop for plastics products, especially in the packaging sector. The EU-wide Packaging and Packaging Waste Regulation (PPWR), adopted in 2024, introduced binding requirements to reduce packaging waste, increase recycling rates, and increase the proportion of recycled materials in new products [1]. However, PCR plastics exhibit significant variations in composition due to differences in feedstock sources, collection methods, and recycling processes, which poses technical challenges for product quality and consistency [2,3]. One of the key factors influencing the variations in material composition and quality is the seasonal fluctuation in the input waste stream (not only regarding plastics), driven by changes in consumer behaviour throughout the year [4,5]. The type, quantity, and condition of discarded plastic packaging can vary significantly depending on seasonal trends. For instance, during the summer months, there is an increase in the disposal of beverage bottles and outdoor product packaging, while the holiday season generates higher volumes of food packaging and household containers [6,7]. These variations can include a mix of polymer types, residual contaminants, degradation byproducts, and inconsistent molecular weight distributions, all of which affect material performance [8,9,10,11].

Such compositional heterogeneity directly influences material behaviour during processing, particularly in common manufacturing methods like injection moulding and extrusion. In injection moulding, variations in melt flow properties, thermal stability, and compatibility of polymer blends can lead to inconsistencies in part quality, such as warpage, phase separation, poor surface finish, or mechanical property deviations [12,13,14]. Similarly, in blown-film extrusion, differences in melt viscosity, elasticity, and molecular structure affect film thickness uniformity, bubble stability, and overall mechanical performance [15,16,17].

HDPE is widely used in applications such as milk jugs, detergent bottles, shampoo containers, and household chemical packaging, all of which exhibit different disposal patterns throughout the year [7,18]. The varying exposure to environmental conditions, contamination from residual contents, and differences in collection and sorting efficiency further contribute to inconsistencies in recycled HDPE quality [18]. Key properties such as melt flow rate, impact resistance, and colour can fluctuate due to the diverse sources of post-consumer HDPE, requiring careful processing adjustments [19,20,21]. To address these challenges, advanced sorting technologies, effective cleaning processes, and controlled blending of recycled HDPE batches are essential to ensure consistent material performance in new products [22,23,24,25]. Despite these efforts, manufacturers using PCR HDPE must continuously adapt formulations (e.g., by using stabilisers or compatibilisers) and processing parameters to compensate for variations, highlighting the complexity of integrating recycled plastics into a circular economy [26,27,28,29,30,31,32].

Understanding the relationships between PCR plastic composition, their processing behaviour, and the resulting product properties are critical for optimising manufacturing conditions, improving product performance, and ensuring reproducibility. The challenge at this point is that the corresponding relationships, especially with regard to polymer blends, fillers, and additives, are not fully understood, even for virgin materials [33,34]. For example, the addition of other components to the polymer either promotes or slows down crystallisation [35]. Furthermore, different ingredients influence the degradation of plastics and thus the molecular structure, which in turn influences the mechanical properties in addition to the processing [36,37,38]. Therefore, the unknown composition of recyclates and their fluctuations make it a complex challenge to create a uniform material quality and to identify possible applications, including existing challenges.

This study aims to quantify the composition of PCR plastics, to compare the molecular structure of different batches and to analyse their effects on the processing parameters and final product properties in both injection moulding and film extrusion. The comprehensive characterisation and processing of several batches can reveal important correlations to provide a basis for future strategies for enhancing the reliability and applicability of recycled plastics in industrial applications. Therefore, this paper is structured in three parts. First, the analysis of the material batches is conducted. Therefore, chemical, physical, optical, and rheological methods are used to characterise the differences among the batches. Afterwards the materials are processed in blown- and cast-film extrusion as well as injection moulding to evaluate the influence of the batch fluctuations on the process stability and resulting product quality. Finally, the results are correlated to determine the relevant material characteristics for consistent PCR processing.

## 2. Materials and Methods

Due to consumer behaviour, the composition of PCR material differs over the course of the year and the different seasons. In this paper, PCR HDPE from the German municipal waste system (so called fraction 329 or DSD 329 bales) was collected over the course of six months (January to June). The fraction is defined as “rigid, system-compatible plastics articles made of PE with a volume of less than five litres”, e.g., bottles and trays, including secondary components such as closures or labels [39]. The proportion of impurities in the fraction is regulated according to a specification and can be up to 6 m % (e.g., wood, textiles, composite materials, metal parts, or foreign polymers) [40]. The batches were received at equal intervals at the end of each month. All PCR HDPE materials were provided in the form of pellets by the same recycler and produced under stable processing conditions within the batches. The analyses and processing presented were conducted under equal conditions within the same timeframe after all the material batches had been received.

**Figure 1:**
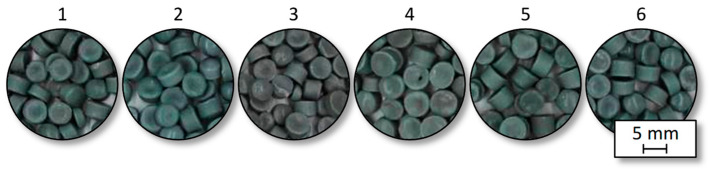
Comparison of the pellet size, shape, and colour of the analysed HDPE batches.

Figure 1 shows pellets from the different batches. To simplify the annotation in the following, the batches are numbered in accordance with their respective months (batch 1 = January). The pellet size and form are similar among the batches and only show slight deviations regarding the colour.

### 2.1. Determination of the Material Composition and Rheology

The different material batches were analysed to determine the composition regarding the amount of inorganic contaminants as well as the foreign polymer content (e.g., polypropylene (PP)). To quantify the inorganic content, ashing tests were conducted in an Heraeus incineration oven type K1253-KAT (Heraeus Holding GmbH, Hanau, Germany). For every batch, three pellet samples (approx. 15 g) were incinerated at 600 °C for 1.5 h. The weight difference before and after incineration allowed for the calculation of the ash residue and, accordingly, the inorganic content in the recyclate material. The ash residue was additionally characterised using a SIGMA VP scanning electron microscope (Carl Zeiss AG, Oberkochen, Germany) equipped with an EDX detector X-max 150 (Oxford Instruments plc, Abingdon, Oxfordshire, UK) to conduct energy dispersive x-ray spectroscopy (EDX). To further understand the batch compositions, the pellets were analysed via differential scanning calorimetry (DSC) and high-temperature gel permeation chromatography (HT-GPC) to estimate the amount of foreign polymers like PP and determine the molar mass distribution in the different batches.

DSC measurements provide important insights into the melting and crystallisation behaviour of polymeric materials, which are crucial for processing and the resulting product properties (e.g., mechanical properties, shrinkage). The melting temperature (T_M_) defines the processing range, while the crystallisation temperature (T_C_) and the associated enthalpies allow conclusions to be drawn about the cooling kinetics, the solidification process, and the degree of crystallinity [41,42,43]. In PCR material, shifted T_M_ or T_C_ values may indicate foreign polymers or contaminants that, for example, increase the melting point through intermolecular interactions and act as uncontrolled nucleation sites. In addition, a higher degree of crystallinity can also lead to a higher T_M_ [44,45,46]. The degree of crystallinity is usually determined by the ratio of the measured specific melting enthalpy to the specific melting enthalpy of a fully crystalline reference material. However, it is almost impossible to determine the degree of crystallinity in an unknown polymer mixture such as PCR material, as the crystallinity of the individual components is also unknown and they interact with each other [47]. Another factor is the presence of crystallisation nuclei such as additives, which influence not only the number but also the size of the crystals formed [48]. The amount of interfacial energy released during the melting of PCR depends on the structure of the crystalline regions, with relatively more energy being released in a fine spherulite structure than in a coarse spherulite structure [49]. In addition, for example, in shorter molecular chains, the number of chain ends increases as a result of degradation, leading to a higher defect concentration and thus a lower degree of crystallisation [50]. To compare these effects for the different batches, DSC analyses were conducted using a DSC Q2000 (TA Instruments—Waters LLC, New Castle, DE, USA) with a temperature range set to −80 °C to 200 °C (first heating) resp. 300 °C (second heating) to ensure the possible characterisation of high-melting polymers such as polyamide (PA) or polyethylene terephthalate (PET) [51]. The heating and cooling rate was set to 10 °C/min. Samples weighing 8 mg were prepared from three different pellets for each batch.

HT-GPC was further used to describe the molecular structure of the batches. In the case of polyethylene, the primary structure describes its constitution, which in turn is characterised by the number, type, and arrangement of the repeat units of a polymer, the type of end groups, and the frequency and length of the branches that make up the macromolecule [52]. In general, the constitution can be described by the molar mass distribution, the weight average molar mass, long-chain branching (LCB), and short-chain branching (SCB) and the SCB distribution, whereby branching plays a less important role for HDPE than for other PE types. All high HT-GPC analyses were carried out using an instrument from PolymerChar, Barcelona, Spain. 1,2,4-Trichlorobenzene (TCB) from Merck KGaA, Darmstadt, Germany, stabilised with 250 ppm butylated hydroxytoluene (BHT), was used as the solvent. Three coupled columns of the type POLEFIN linear XL with a particle size of 20 µm and a pre-column of the type POLEFIN with a particle size of 20 µm from Agilent, Santa Clara, CA, USA, were used. The samples were dissolved in TCB in an oven at 160 °C for 90 min before injection (approx. 200 µL solvent). The sample quantity used was 16 mg ± 1 mg. To reduce measurement fluctuations, 1 mg to 3 mg BHT was added as an internal standard during the preparation of each sample before dissolution. The HT-GPC was equipped with a multi-band infrared (IR) detector, model IR6, and a 4-capillary viscometer, both from Polymer Char. Narrow polystyrene standards, also from Polymer Char, were used to calibrate the HT-GPC. All data were analysed using the PSS WinGPC UniChrom software V8.40 from Agilent.

As the resulting part warpage due to shrinkage after processing is determined by the pvT-behaviour of the specific material, the temperature- and pressure-dependent specific volume was additionally determined using a PVT 500 (also Göttfert Werkstoff-Prüfmaschinen GmbH, Buchen, Germany). The used capillary had a length of 10 mm and a diameter of 1 mm. The recyclate batches were measured at five different pressures (200, 400, 800, 1200, 1600 bar). The pellets were heated up to 240 °C, and the change in specific volume was measured under constant cooling (5 °C/min). Due to the long testing duration, single measurements were conducted.

To correlate the processing behaviour with the material viscosity, the rheological properties were determined using conventional melt flow rate (MFR) measurements as well as process-oriented high-pressure capillary rheometry (HCR). The MFR measurements were conducted on a Meltflixer HT (Thermo Fisher Scientific Inc., Karlsruhe, Germany) based on DIN EN ISO 1133-1 [53]. Three measurements were conducted for every batch at 190 °C and 230 °C and a static weight of 5 kg due to the low MFR range provided in the material data sheet. Additionally, the MFR was measured before and after drying (3 h, 80 °C) to determine the influence of the drying process on the material viscosity. Since the process conditions regarding shear velocities and temperatures vary for injection moulding and extrusion processes, the HCR measurements were carried out for two different shear velocity areas and temperatures (190 °C and 230 °C) on a Rheometer 2002 (Göttfert Werkstoff-Prüfmaschinen GmbH, Buchen, Germany). The shear velocities were selected to represent conditions during injection moulding (stamp speeds: 2.78, 1.39, 0.56, 0.28, and 0.14 mm/s, resulting in rep. shear rates of approx. 4075, 2038, 815, 408, and 204 s^−1^) as well as extrusion (stamp speeds: 0.56, 0.28, 0.14, 0.06, and 0.03 mm/s, resulting in rep. shear rates of approx. 815, 408, 204, 82, and 41 s^−1^).

### 2.2. Processing Through Extrusion and Injection Moulding

The focus of design for recycling is shifting towards mono-material packaging, which leads to the necessity of the same materials being applied to different products and that are thus produced through formerly more uncommon processing techniques. Among other things, this leads to PE films being combined with injection-moulded components, such as mono-PE pouches, with the result that HDPE is not only used in blow-moulded packaging anymore [54]. To fully understand the interactions between the material composition and resulting product properties, the batches were therefore processed using blown- and cast-film extrusion as well as injection moulding. This enabled a differentiated analysis of the material behaviour.

Although HDPE is not typically processed as a monofilm but only in layers in blown-film extrusion, the high requirements for processing thin films, in contrast to thermoforming or blow moulding, enable more accurate characterisation of the material and an inline measurement of small fluctuations in processing and the resulting product properties. The PCR HDPE materials were therefore first extruded into a monofilm on a blown-film line from Kuhne Group, St. Augustin, Germany. An extruder with a configuration of 45 mm and L/D = 24 was used, as well as a die with an outlet diameter of 80 mm. The die gap was set to 1 mm, the blow-up ratio to 2.4, and the output to 10 kg/h to reach the desired film thickness of 100 µm to 200 μm. The die, or rather, the die head temperature was set to 230 °C. With these moderate process parameters, the aim was to compare the processability of the different batches in the (especially for PCR materials) demanding blown-film extrusion process. A challenge when processing recycled materials into films, during further processing (e.g., laminating, printing) and in terms of the resulting product properties (e.g., protective functions), is the occurrence of gels. Gels are small visual defects or elevations in the film due to isolated impurities. In industry, modern production plants are often equipped with optical inspection systems to ensure continuous in-line quality control. Since the blown-film extrusion line did not have such a system, the PCR materials were processed on a cast-film line from OCS Optical Control Systems GmbH, Witten, Germany, as part of a quantitative investigation into the influence of gels at constant process parameters. The die temperature was set to 210 °C, with a screw speed of 30 rpm. The system had an integrated film surface analyser FSA100 for detecting optical defects, which used a 3CMOS line scan camera with LED lighting [55]. Approximately three metres of film per minute were measured for the investigations. In practice, corresponding laboratory systems are connected directly to the production process, for example, at the production sites of raw material manufacturers, to monitor the quality and homogeneity of materials precisely and at an early stage. In order to verify the extent to which a reduced gel number influences processing, the recycler provided another batch. This material is referred to below as the ‘high-purity batch’ and was additionally processed after the usual recycling process, with an additional filtration stage being used on a laboratory scale.

**Figure 2:**
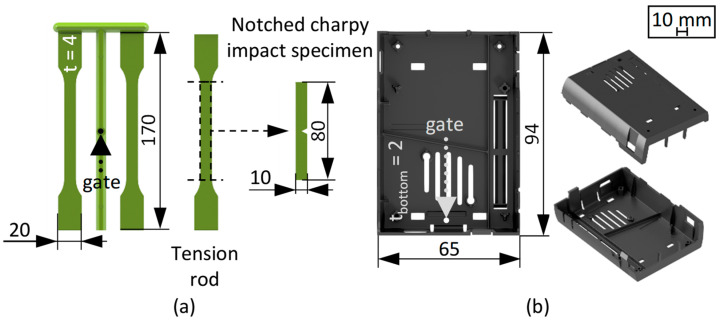
Dimensions and thicknesses (t) of the injection-moulded parts. Tension rod and milled notched Charpy specimen (**a**), casing geometry (**b**). The gate positions are marked with dotted arrows.

The injection moulding process was carried out using an all-electrical Sumitomo SHI Demag IntElect2 100/470-250 machine (Sumitomo (SHI) Demag Plastics Machinery GmbH, Schwaig, Germany) with a 30 mm screw diameter and 100 t clamping force. To evaluate the mechanical properties as well as the effects of processing on the geometric and optical part quality, two distinct geometries were selected. A cold runner two-cavity injection mould was used for manufacturing type 1A tension rods based on DIN EN ISO 527-2 (Figure 2a, [56]), while a hot runner mould was utilised to produce a more complex casing geometry (Figure 2b).

Both moulds were cooled via water temperature control units of type Thermo-6 (HB-Therm AG, St. Gallen, Switzerland). Out of the two produced tension rods, one was used to conduct tensile tests, while the other was milled to create a type 1 notched Charpy impact specimen according to DIN EN ISO 179-1 (notch type A, 45°) [57]. To validate the material fluctuations, the tension rods were produced at two different cylinder temperatures. The casing geometry was only produced at the higher temperature due to the thinner wall thickness and high viscosity of the material. The relevant injection moulding process settings are given in Table 1.

The injection moulding machine’s control system provides access to Open Platform Communication Unified Architecture (OPC UA) interfaces, allowing OPC UA clients to monitor the process and retrieve cyclical data, such as the maximum injection pressure, maximum torque, melt cushion, and dosing time. Beyond cyclical data, continuous process data can be collected and extracted using a machine-specific Data Acquisition System (DAS) Light. This system enables the visualisation of individual production cycle data in curves—such as the injection pressure, screw position, rotational speed, and torque. The data is stored, downloaded, and analysed using VIPRA^®^ by SHS plus GmbH, Dinslaken, Germany.

### 2.3. Offline Product Quality Analysis

The injection-moulded tension rods were mechanically analysed. Therefore, tensile tests were conducted based on DIN EN ISO 527-1 [56] using a ZwickRoell Z100 testing machine (ZwickRoell, Ulm, Germany) equipped with a MultiXtens extensometer (ZwickRoell) and a U3 load cell (HBM Machines B.V., Moordrecht, The Netherlands). The modulus of elasticity was initially measured at a speed of 1 mm/min. The test then proceeded at a speed of 50 mm/min until either the specimen broke or an elongation of 300% was reached. To determine the Charpy notched impact strength based on DIN EN ISO 179-1 [57], a Zwick 5101 impact pendulum (ZwickRoell) was used. The width of the supporting span was 62 mm. The impact force was applied by a 2 J hammer with a friction work of 0.01 J. The corrected impact energy *W*_*C*_ in J was calculated according to Formula (1). *W*_*U*_ refers to the uncorrected impact energy in J, and *W*_*R*_ refers to the friction work in J.

*W*_*C*_ = *W*_*U*_ − *W*_*R*_ (1)

The Charpy notched impact strength *acN* in kJ/m^2^ was determined according to Formula (2). *W*_*C*_ indicates the corrected impact energy in J, *h* the thickness of the specimen in mm, and *bN* the residual width of the specimen in the notch base in mm.

(2) $$a_{cN}=\frac{W_{c}}{h\times b_{N}}\times 10^{3}$$

The consistency of the complex part’s geometry was also evaluated. To assess dimensional differences between the batches, two transverse and two longitudinal spans were measured using a GOM ScanCobot equipped with an ATOS Q optical 3D scanner (both Carl Zeiss GOM Metrology GmbH, Braunschweig, Germany). The scanner created a complete 3D-model of the real injection-moulded part. Afterwards, the dimensional deviation was determined by comparing the defined measurement spans of the injection-moulded casings with a 3D reference geometry of the casing part, which was used to design the injection mould. Since the same reference was used for all batches, the dimensional deviations among the batches can be compared and correlated with different material properties.

## 3. Results and Discussion

First, the material properties of the analysed batches are shown, followed by a study on processability and the mechanical and optical properties of the products. Due to the diversity and extent of the conducted analyses, the presented results are directly discussed and interpreted, which allows for referencing and the presentation of connections among the results. To conclude, the results are statistically evaluated to determine composition–property correlations relevant for ensuring batch quality stability using Pearson’s correlations. The sample size for the corresponding data sets or analyses is given in the presented diagram. If the sample size is >1 and no error indicators are recognisable, the standard deviations are presented but are minimal and therefore not visible.

### 3.1. Material Analysis

The different conducted material analyses allow for correlations between the material composition and the resulting viscosity and mechanical behaviour. The challenges of the interpretation of the results lie within the complex interactions between foreign polymers and contaminants as well as degradation effects in PCR, which lead to changes in the molecular weight (distribution) and crystallisation behaviour.

#### 3.1.1. Residual Ash Content

**Figure 3:**
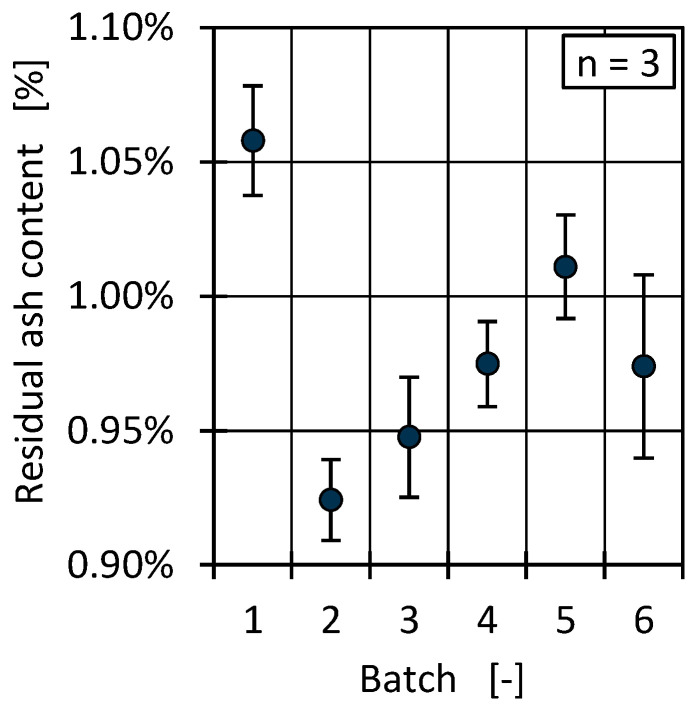
Residual ash content for the different material batches.

In contrast to virgin material, all the analysed HDPE PCR batches showed a residual ash content. Since the incineration was conducted at 600 °C, the residue only consisted of inorganic particles. Figure 3 shows the determined residual ash contents for all batches.

**Figure 4:**
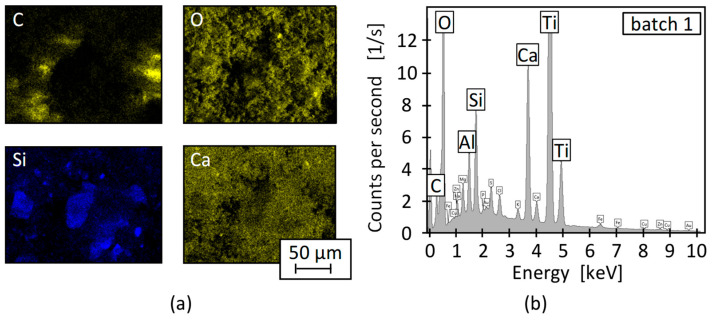
Exemplary element mapping (**a**) and resulting element frequency (**b**) for batch 1.

The ash content varied between 0.9% and 1.0%, with a maximal deviation of 14% between batch 1 and 2. For HDPE PCR, a residual ash content around 1% lies within a lower market typical range, indicating a low inorganic contamination level [9,58]. The composition of the ash residue was determined via EDX measurements. The results show high contents of oxygen, titanium, calcium, carbon, silicon, and aluminium, which suggest the inorganic materials were at least partially due to pigments from labels or colours in the form of titanium dioxide or additives such as calcium carbonate [9,59]. An exemplary element mapping (a) as well as the resulting EDX spectrum (b) is given in Figure 4 for batch 1.

#### 3.1.2. Pressure- and Temperature-Dependent Specific Volume (pvT)

**Figure 5:**
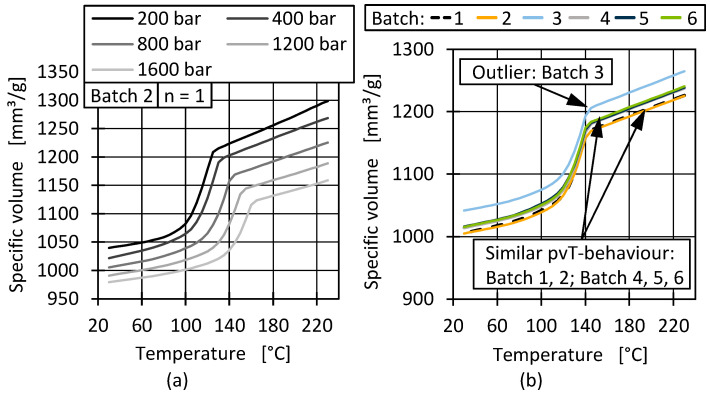
Pressure-dependent pvT behaviour of batch 2 (**a**) and compared pvT behaviour of all batches at 800 bar (**b**).

During injection moulding, polymers undergo a defined pressure and temperature progression, which results in different changes in specific volume depending on the material. Figure 5a shows the exemplary pvT behaviour of batch 2 for five pressure levels. The curve progressions are typical for a semi-crystalline material and similar for all batches.

To determine differences between the batches, Figure 5b displays the specific volume over the temperature for all batches at 800 bar, which is a relevant pressure area for both produced parts in injection moulding. However, due to the simple measurement method and the significant outlier behaviour of batch 3, it is unclear whether the results accurately represent the batches. The levels in Figure 5b therefore only suggest that the batches exhibit different pressure dependencies. This could lead to differences in shrinkage during cooling in injection moulding and extrusion and therefore influence, for example, the film quality and the warpage of injection-moulded parts [60,61,62].

#### 3.1.3. Crystallisation and Composition Regarding Foreign Polymers (DSC)

**Figure 6:**
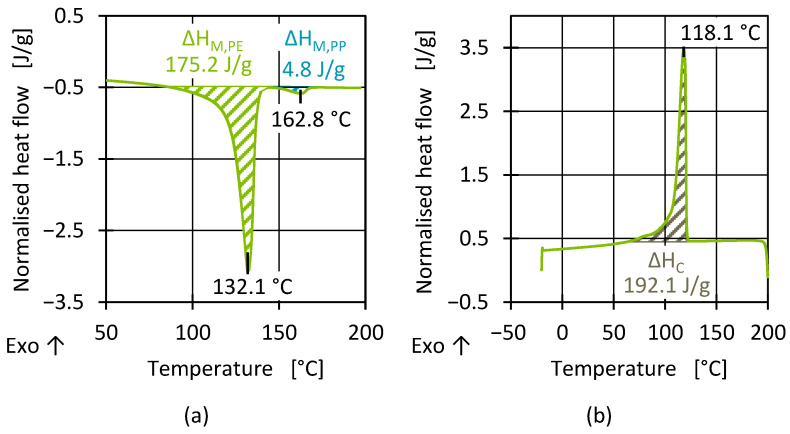
Representative DSC heating curve with two enthalpy peaks (**a**) and cooling curve (**b**) for batch 2.

To verify batch consistency, the melting and crystallisation behaviours were determined using DSC. Figure 6 shows representative heat flows of the second heating and first cooling for a sample from batch 2. When determining the melting enthalpy, it is evident that, in addition to a PE melting peak at around 130 °C, all of the analysed batches displayed a further peak at typical PP melting temperatures (around 160 °C) [63]. The heat flows for temperatures higher than 200 °C are not shown, because no further peaks could be identified. However, this does not mean that the recyclate did not contain any thermoplastic polymers such PA or PET but rather that the possible quantity contained was below the detection limit of the DSC device in relation to the sample quantity. For this reason, FT-IR measurements were also conducted on the batches. However, the results also showed no unusual peaks, except for indications of PP contamination. Typical barrier materials and other foreign polymers could not therefore be detected. The PP content was quantified using IR, as shown in Section 3.1.4, in combination with HT-GPC measurements.

A secondary melting peak was present in all conducted measurements but varied in size (Figure 6a). This suggests that the different batches showed a different melting behaviour or contained different proportions of PP. With the current state of technology, completely separating PE and PP in the mechanical recycling process is a challenge, also due to the similar density, which often leads to PP contamination in PE. To estimate the PP content in the recyclates, measurements were carried out, in particular using Fourier-transform infrared spectroscopy (FT-IR) and DSC, as these methods are readily available and offer fast analysis times [28,37,47,64,65,66,67]. Still, the results of both analytical methods were highly dependent on the used PP and PE types.

The evaluation of the crystallisation poses greater challenges. In contrast to the melting curve, no additional peak was determined during the cooling (crystallisation) of the batches (Figure 6b). While the PP content in the PCR material melted separately from the PE matrix, the cross-contamination influenced the overall crystallisation kinetics of both materials, which led to an overlap of the crystallisation enthalpies. Due to the linear, regular structure of the polymers, the crystallisation temperatures of HDPE and PP were close to each other and the materials crystallised simultaneously [68]. The presence of foreign polymers combined with potential variations in the batches with regard to molar mass distribution, nucleation effects (e.g., additives or PP), and the presence of small amounts of foreign polymers or inorganic components further affected the crystallisation process compared to that of pure polymers. In addition to the different polyolefin (PO) types, the proportion of components and other impurities (e.g., additives) also influenced the resulting size, shape, and spatial distribution of the formed polymer crystals, as well as the degree of crystallinity. This is because the components interacted with each other despite a lack of miscibility. As a result of the unknown complex composition of the recyclates and due to the different sources, up to this point, it was a challenge to predict the morphology and the resulting physical properties of the PCR material without knowledge of the corresponding waste stream. Important conversion temperatures and enthalpies were therefore determined to quantify batch fluctuations and to derive possible causes.

To assess the differences in melting and cooling behaviours, Table 2 shows the measured melting temperatures during the second heating and crystallisation temperatures during cooling of the tested HDPE batches. A comparison of the conversion temperatures shows that the differences for both temperatures were less than 1 °C. Therefore, an exclusive consideration of the melting and crystallisation temperature does not indicate significant differences in thermal behaviour [69].

**Figure 7:**
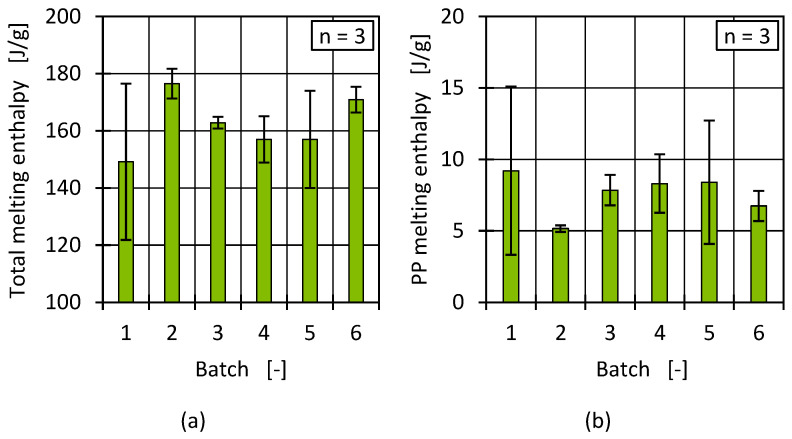
Determined total melting enthalpy (**a**) and PP melting enthalpy (**b**) for all batches.

Apart from the process-relevant transition temperatures, the melt enthalpies of the batches were analysed. The total enthalpy and the PP enthalpy were determined for each batch and measurement. The results are presented in Figure 7. The significant deviations in the enthalpies indicate a high degree of inhomogeneity in the material, at least in relation to the sample size used for a DSC measurement. The batches varied among themselves and to some extent within a sample. For example, batches 1 and 5 showed large fluctuations in both the total enthalpy and the PP enthalpy within the material. These could be influenced, for example, by individual larger PP phases in a pellet. Further differences between the batches were particularly evident for batch 2, which had a high total melt enthalpy and a lower PP enthalpy compared to the other batches.

**Figure 8:**
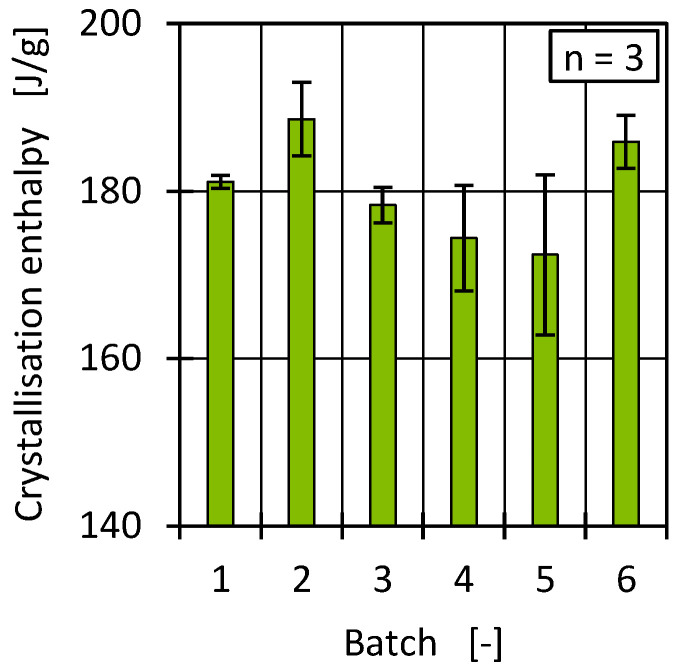
Determined crystallisation enthalpies for all batches after first heating.

Since high fluctuations complicate definite interpretation, the crystallisation enthalpy was further analysed to determine to what extent the enthalpy results for the batches were reflected by the PP content and/or to what extent a higher degree of crystallinity was present and how this affected the material properties. In contrast to the melt enthalpy, the crystallisation enthalpy represents the heat released when a polymer (in this case, HDPE) transitions from a molten state back into a solid crystalline state through an exothermic process. Typically, crystallisation occurs/starts at temperatures below the melting temperature [70,71]. Figure 8 shows the crystallisation enthalpies for all batches.

Similar to the results of the melting enthalpy, batches 2 and 6 had a higher enthalpy of crystallisation than the other batches. An increased PP content in HDPE can lead to a reduction in the crystallisation enthalpy [69], so by comparison, an increased crystallisation enthalpy, compared to the other batches, indicates a reduced PP content in batches 2 and 6, in line with the results of the melting enthalpy of PP (see Figure 7).

#### 3.1.4. Molecular Structure (HT-GPC)

**Figure 9:**
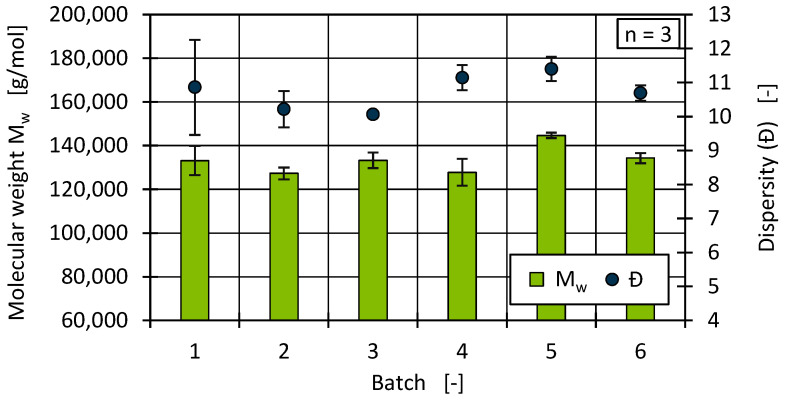
Average molecular weight and dispersity for all batches.

In addition to the processing conditions, such as the cooling rate and the orientations that arise during the plastic processing, the crystal morphology is largely determined by the primary structure of the material [45,72,73]. In the following, the molar mass and its distribution are therefore considered by means of HT-GPC. Figure 9 shows the average molecular weight (MW) and the dispersity (Đ) for the PCR HDPE batches.

**Figure 10:**
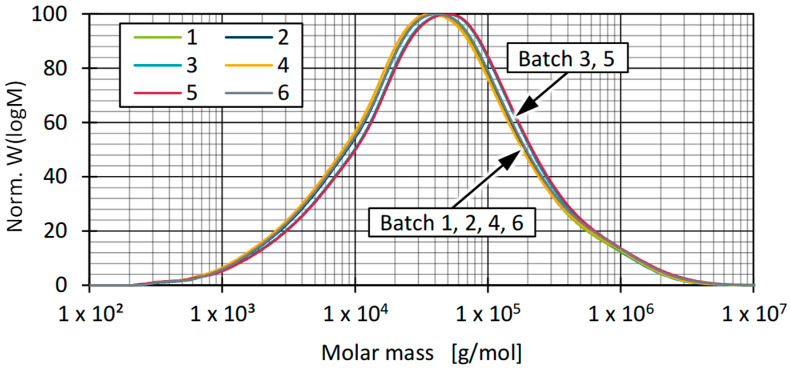
Median of the molecular weight distribution for all batches.

Overall, the determined molecular weights were on the same level, with only batch 5 showing a significantly higher MW value than the other batches. It was also noticeable that the Đ for batch 1 showed strong fluctuations, and no clear outlier could be identified in the three molecular weight distributions determined. Overall, the Đ also fluctuated between batches, although the fluctuations were not related to the median of the molecular weight distributions (see Figure 10).

The distributions show that, in principle, the lower and upper molecular weight limits corresponded, but the distribution maximum shifted to higher molar masses for individual batches. The median of batch 3 and batch 5 showed a strong similarity, whereas the mean values tended to indicate a comparable distribution between batch 4 and batch 5. This finding suggests that the composition of the HDPE materials varied greatly and that the analysis results were characterised by larger outliers. Likewise, the differences in the distributions show that the Đ alone is insufficient as a scalar value to describe the distribution, since it does not contain any details about the exact shape and position of the distribution. Since only three HT-GPC analyses were carried out for each material, it was necessary to examine the extent to which the median as an average distribution provides an approximately representative statement.

**Figure 11:**
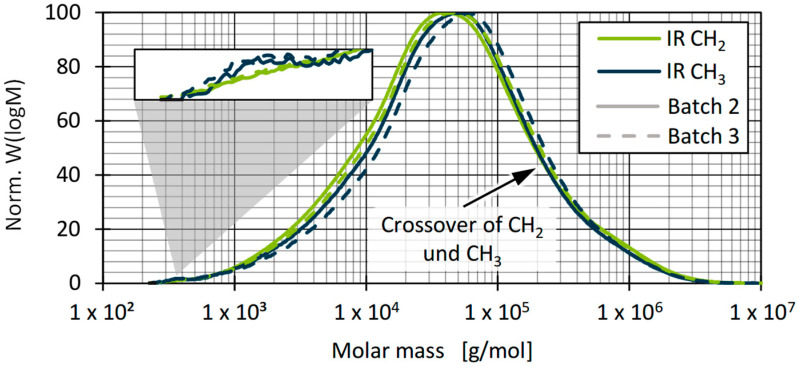
Normalised weight distribution of CH_3_/CH_2_ content for batches 2 and 3.

For further analysis of the recyclates, a distinction was made between CH_2_ and CH_3_ groups to describe the influence of PP [74,75]. For this purpose, the ratio of narrow filter bands in the range of the stretching vibrations CH_3_/CH_2_ was used to conclude an increasing PP content based on the increase in CH_3_ groups. The advantage of using HT-GPC-IR is that the polymer in solution retains its structural order, and a distinction between amorphous and crystalline areas can be eliminated. The applied analysis is usually used to quantify the SCB. In this context, it was therefore assumed that the influence of the SCB and the influence of other foreign contaminations containing CH_3_ groups could be quantitatively subordinated to the PP. Corresponding distributions for batch 2 and batch 3 are shown in Figure 11.

A comparison of the CH_2_ and CH_3_ distributions shows that they crossed over towards higher molar masses, and thus, PE predominated over PP in the high-molecular-weight range. This seems logical, in that rigid HDPE waste consists mainly of blow-moulded products, while rigid PP packaging is produced either by extrusion, blow moulding, thermoforming, or by injection moulding. Since lower viscosities are generally required for injection moulding and the corresponding materials usually have significantly lower molar masses, the PP components in the mixture indicate a lower number of very long chains but a nevertheless very balanced use of the various processing methods. This observation is consistent with the findings of sorting analyses conducted by other researchers, which showed an approximately 50:50 distribution of products produced by the different processing methods in the composition of PP bale goods [76].

Furthermore, it was observed that a small shoulder formed in the low-molecular-weight range, which cannot be assigned to PE, since the corresponding signals primarily refer to CH_3_ groups. These could be assigned to additives contained in the material, e.g., stabiliser systems or slip agents, which typically have a molecular weight between 200–600 g/mol or low-molecular-weight PP [77,78]. Due to the presence of CH_3_ groups in both the low-molecular-weight and high-molecular-weight range, as well as the shift in the distribution maximum, only assumptions can be made about influences on the viscosity.

**Figure 12:**
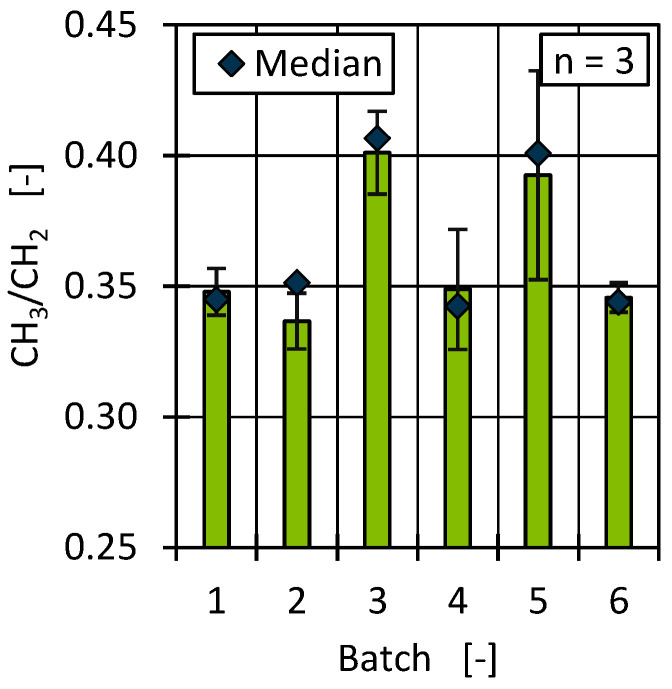
CH_3_/CH_2_ ratio as a measure of the PP content in recyclates.

A quantitative determination of the PP proportions in the recyclates cannot be made based on the comparison of the CH_2_ and CH_3_ groups. Nevertheless, based on the determined CH_3_/CH_2_ ratios in relation to the entire distribution range, an estimation can be made as to which batches tended to contain more or less PP. Figure 12 shows a comparison of the occurrence of PP, represented by the CH_3_/CH_2_ ratio. The results show comparable proportions of PP for batch 3 and batch 5, which tended to be higher than for the other batches. The proportions of PP thus correlated with the shape of the molecular weight distribution, whereby the composition of the examined batches, independently of the respective proportion of PP, showed a high degree of similarity overall (see Figure 10). Furthermore, the median appears to be a good basis for comparing the molecular weight distributions by considering a total of three samples.

#### 3.1.5. Melt Flow Rate (MFR) and Representative Viscosity (HCR)

**Figure 13:**
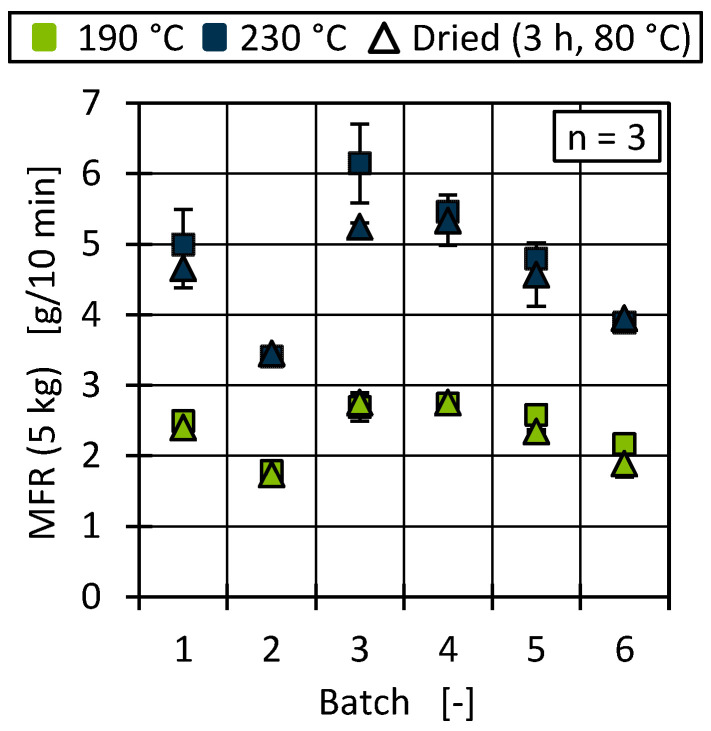
MFR data for all batches measured at 190 °C and 230 °C with and without previous drying.

The flowability of thermoplastics is usually characterised by means of the MFR in data sheets or as part of an incoming goods inspection before being passed on to production [79,80]. This characteristic value is used to evaluate and ensure the processability of the material in injection moulding or extrusion. However, the disadvantage of the MFR is its limited informative value regarding processing-relevant shear rates. Due to this limitation, both the MFR value and the shear viscosity were measured for relevant temperatures and shear rates in this publication. Figure 13 shows the MFR results for all batches at two temperatures depending on the drying condition.

At 190 °C, the MFR varied by 53% from batch 2 (1.79 g/10 min) to batch 4 (2.74 g/10 min). The increase in measurement temperature also increased the MFR and resulted in a maximum difference of 2.74 g/10 min (batch 3 vs. batch 2). Due to the overall low MFR for processing in injection moulding [59], the changes in viscosity could lead to instable processing conditions. Figure 13 also shows the influence of drying on the resulting MFR. Virgin HDPE is a non-hygroscopic material, and drying is not necessary before processing. However, contaminants (e.g., paper, label residues) and hygroscopic foreign polymers (e.g., PA, PET) can lead to hygroscopic material behaviour that is atypical for polyolefins [81]. Drying showed no significant influence on the measured MFR at low temperature for all six batches. At 230 °C, drying tended to lead to a decrease in MFR (especially for batch 3), which could be a result of slip-additive-like properties of water during testing at a higher temperature.

**Figure 14:**
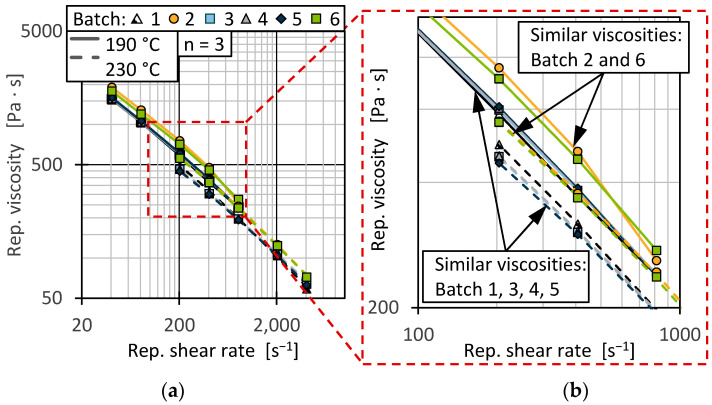
Representative viscosities over representative shear velocity at 190 °C and 230 °C. For all shear rates (**a**) and a magnified shear rate range (**b**).

To evaluate the flow behaviour under process-related conditions, the material viscosity was determined using HCR. Figure 14 shows the resulting viscosity curves for the temperatures and shear rate ranges for extrusion and injection moulding.

The curve progression for all batches was comparable. However, batches 2 and 6 showed a shift towards a higher viscosity over the measured shear rates for extrusion as well as injection moulding. This correlated with the lower measured MFR for those batches shown in Figure 13. The higher temperature during the measurement for injection moulding conditions resulted in a shift of all curves towards a lower viscosity [79,82], also aligning with the shift towards a higher MFR at an increased temperature. The differences in viscosity were more prominent for the lower temperature.

Differences in material viscosity can be attributed to various causes. Particularly in the case of recyclates, it should be emphasised that interactions between, for example, foreign polymers and impurities or degradation mechanisms of polymers and additives due to reprocessing make precise interpretation more complex [9,10,83,84]. As Figure 13 and Figure 14 show, the viscosities of the batches can be divided into two ranges, with batches 2 and 6 as well as 1, 3, 4, and 5 behaving similarly. Comparing this with the results of the DSC and HT-GPC measurements, hypotheses can be derived that help to explain the differences in rheological behaviour. One explanation for the higher viscosity (and the correspondingly lower MFR) of batches 2 and 6 could be the lower PP content. PP components in rigid packaging are often manufactured from low-viscosity injection moulding grades and can therefore influence the overall viscosity of the HDPE recyclate depending on the PP content [11]. One possibility is to derive the relatively low PP content from the lower PP enthalpy (DSC, see Figure 7) as well as the overall higher melting and crystallisation enthalpy (DSC, see Figure 7 and Figure 8) [67,85]. Another possibility can be derived from the results of the CH_3_/CH_2_ ratio from the HT-GPC analyses, which differed somewhat, indicating differences between batches 3 and 5 compared to 1, 2, 4, and 6 (see above, Figure 12). It is therefore possible that the low-molecular-weight shoulder in the molecular weight distribution (see Figure 11) was actually due to additives like slip agents that have a significant influence on flow behaviour. For example, octadecanoic acid (stearic acid) and octadecanamide (stearamide) can be assigned to the corresponding molar mass range, which generally exert a strong effect on wall slip [77]. In addition, fluoropolymer-based processing aids, which are currently being discussed but still used, not only influence the process itself but also the melt viscosity. Such additives are of a high molecular weight and contribute, among other things, to reducing energy consumption in the extruder and minimising deposits and surface defects. Compared to conventional lubricants, these are only used in very low concentrations [86]. Therefore, hardly any change is expected in relation to the molecular weight distribution of the recycled sample. In addition to fluoropolymers, polyethylene glycol (PEG) is used as a synergist to improve the distribution of processing aids on surfaces such as extrusion nozzles [87,88]. While fluoropolymers only have a slight effect on the crystallinity of the polymer, PEG has a complex effect on crystallite formation [89,90]. It is therefore possible that not only the viscosity but also the crystallinity and thus the apparent PP content according to the DSC is influenced by the additives.

### 3.2. Influence of Batch Fluctuations on the Process Stability and Film Morphology During Blown- and Cast-Film Extrusion

**Figure 15:**
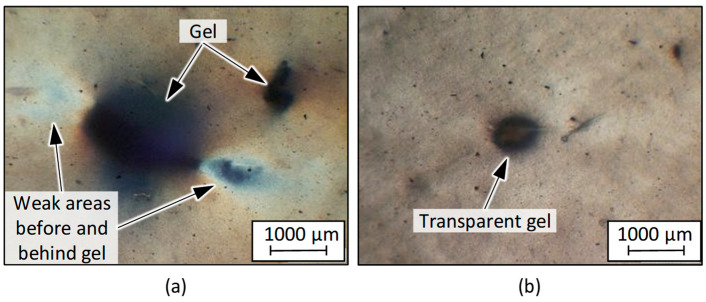
Examples of gels in extruded blown film of batch 1, highlighting weak areas (**a**) and a transparent gel (**b**).

The batches were processed into blown films under the described constant process parameters. The processing initially showed good bubble formation and good bubble stability. This behaviour can be partly attributed to the comparatively broad molecular weight distribution with a dispersity of about 8–9. Despite the overall good processing behaviour, no stable process was possible with the PCR HDPE batches. During the blowing process, the film tube tore due to the impurities or gels contained in the material. The number and size of these gels in the recyclate were so high that the extrusion process could not be run smoothly even for a period of 30 min. Figure 15 shows two examples of gels in the extruded film of batch 1, created through transmitted-light microscopy. All gels initially appeared dark because they were surrounded by the grey recyclate matrix during the extrusion process. On closer inspection, however, it can be seen that only the gels in Figure 15a are actually dark or black, while the gel in Figure 15b is light to transparent. In-depth investigations of individual gels have already been carried out in the past on film recyclates using a heating table in combination with FT-IR [36]. Dark gels could be attributed in particular to rubber or oxidised PE types. Light-coloured gels, on the other hand, could often be traced back to (partially) cross-linked PE or corresponding copolymers such as ethylene vinyl acetate (EVA). A similar behaviour is suspected for the PCR HDPE shown here, but this has not been investigated in more detail.

Regardless of the origin of the gels, the images show that weak spots developed primarily in front of and behind large gels, which were likely responsible for the process disturbances. Accordingly, it was not possible to take samples for mechanical testing. It was therefore not possible to analyse possible interactions between the material composition and the resulting film properties. To verify the basic processability of comparable PCR batches without the influence of gels, the ‘high-purity batch’ was processed. The recyclate came from the same waste source and the same recycler but had been refiltered in a further extrusion step to reduce the number of gels. The test confirmed that, with the same process settings, trouble-free processing is possible and that the gels contained in the recyclate represent a central challenge in processing. Accordingly, HDPE recyclates are generally also suitable for film processing, provided that sufficient gels can be removed in the commercial recycling process.

**Figure 16:**
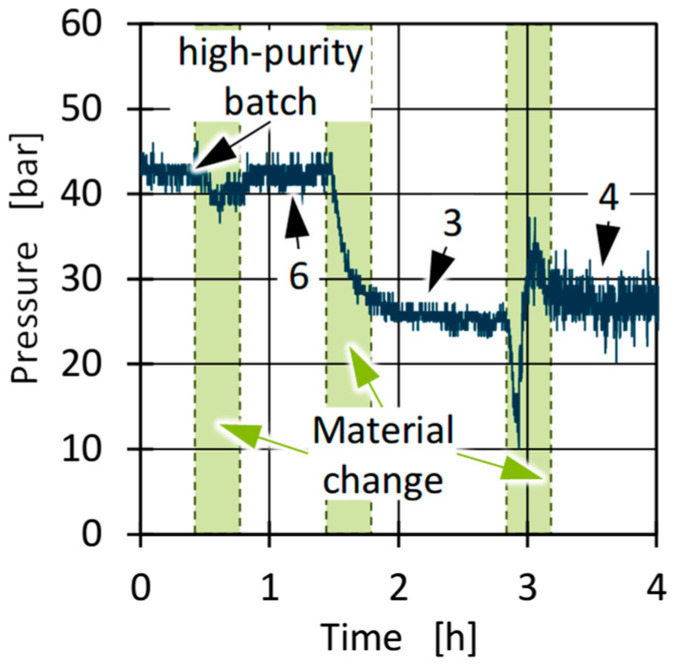
Pressure difference between extruder and die for the high-purity batch and selected PCR HDPE batches 3, 4 and 6 during processing in blown-film extrusion.

To further detect material fluctuations within a batch and between batches in the case of larger material quantities, the pressure between the extruder and the die was measured inline during processing. Figure 16 shows an example of the pressure curve for batches 6, 3, and 5 as well as for the high-purity batch. The curve shows similar pressure levels for the high-purity batch and batch 6 and for batches 3 and 5. The differences between batch 6 and batches 3 and 5 correspond to the results of the viscosity measurements. However, the pressure fluctuations for batch 5 are particularly striking, with pressure differences in the range of about 10 bar. A repetition of the tests on a further test day with the remaining batches 4 and 1 showed comparable pressure levels for batch 4 as for batch 3, while batch 1 exhibited similarly high pressure fluctuations and a similar pressure level as batch 5. Based on the higher pressure for batches 2 and 6 and the lower pressure for 1, 3, 4, and 5, the pressure in the inlet appears to correlate directly with the viscosity of the material. Since significantly more material was used for the blown film tests than for standard HCR measurements, it can be concluded that the used samples for rheological measurements represent a sufficiently representative amount of material to describe the viscosity of the batches. However, the rheological measurements probably cannot adequately reflect fluctuations within individual batches, as detected in particular for batches 1 and 5. A comparison of the observed pressure fluctuations with the standard deviations of the melting enthalpy from the DSC measurements (Figure 7) indicates a possible correlation. This leads to the hypothesis that thermal analyses may be able to visualise internal material fluctuations.

#### Amount of Gels

**Figure 17:**
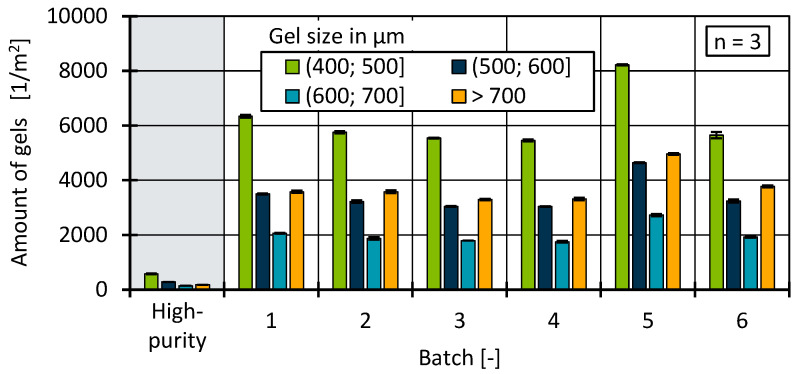
Comparison of the number of gels between a further processed HDPE recyclate and the analysed batches.

Measurements were carried out during the cast film process using the optical inspection system to analyse the number of gels as a function of the batches. A resolution of 25 µm was used during inline inspection. However, only gels with an equivalent circle diameter of 400 µm were considered for final evaluation. This is because the number of impurities increases significantly in the small gel size range. With a size limit of >150 µm, well over 100,000 total defects per square metre were detected for the batches. This resulted in extensive data volumes that are less relevant in view of the current requirement profiles for products made from recycled materials [91]. In addition, similar trends can be observed across all size classes, so that no restrictions on the intended investigations are to be expected by focusing on larger gels. Figure 17 shows the number of gels for all batches examined, divided into four size classes. The results initially showed major differences between the high-purity batch and the other batches and therefore validate that further processing significantly improved the material quality and, in this case, enabled processing through film extrusion. A challenge in this context is the willingness of recyclers to provide information about their specific recycling and filtration processes, reducing possible correlations between processing steps and material quality.

Batch 5 stands out among all batches with a higher number of gels in all size classes. It is interesting to note at this point that batch 5 also showed a higher molecular weight in the HT-GPC measurements. It is therefore possible that batch 5 underwent a stronger chemical degradation process due to thermal–oxidative degradation, resulting in the formation of more cross-linked gels. Various scientific studies on pure substances with several processing steps, i.e., accumulated molecular damage, show cross-linking and branching as the dominant degradation mechanism for polyethylene in a closed cycle [78,92,93,94,95]. Furthermore, the authors were able to show earlier that a varying material composition has a strong influence on gel formation, for example, due to the presence of PP [36]. However, it cannot be determined at this point whether increased chemical ageing due to changes in the process control in recycling or due to a fluctuating material composition is more likely. Nevertheless, depending on the application of the recyclate, gel analysis—either inline or as part of the quality control process—is an important tool for checking the processability of blown films or the stretchability of materials in general.

### 3.3. Influence of Batch Fluctuations on the Process Stability and Part Quality During Injection Moulding

**Figure 18:**
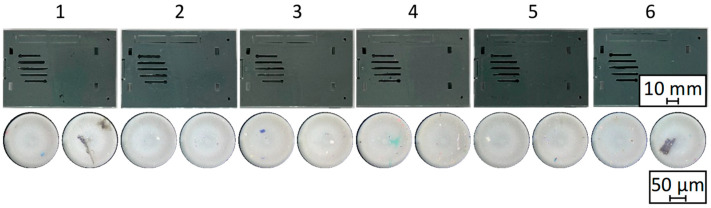
Exemplary part images of the produced casing geometry for all batches (**top**) and additional microscopic surface images (**bottom**).

During injection moulding, the complex casing geometry (see Figure 2) as well as the tension rods were produced under equal conditions for all batches. Exemplary casings are presented in Figure 18 and only show slight deviations in part colour. The exemplary microscopic images were generated with a MICROFLIP pocket microscope (Carson Optical, Ronkonkoma, NY, USA) directly on the part surface and show that (in)organic contaminants were present in different areas of the produced parts from all batches.

**Figure 19:**
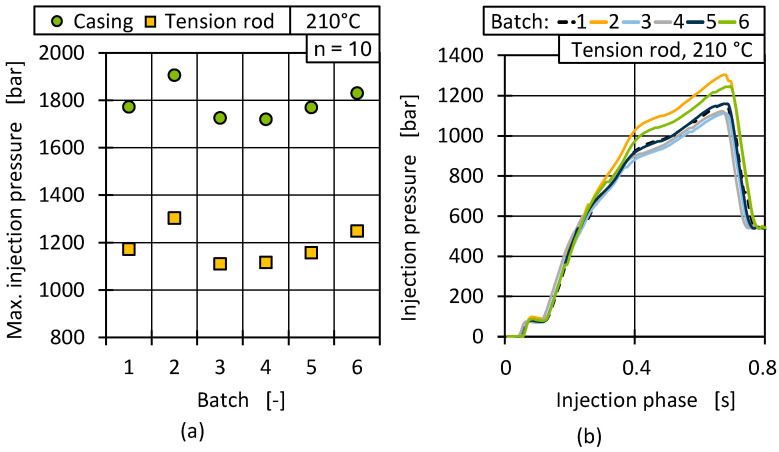
Maximum injection pressure at 210 °C for all batches depending on the part geometry (**a**) and representative continuous pressure curves for all batches (**b**).

To correlate the material characteristics with the injection moulding processing behaviour, process data was analysed in the next step. An industrially commonly used key figure to determine process stability is the maximum injection pressure. It is a cyclical data point typically provided by all injection moulding machines without additional soft- or hardware modifications. Figure 19a shows the maximum pressure during injection for all batches at a 210 °C cylinder temperature.

Due to the lower part thickness of the casing geometry in contrast to the tension rods, the maximum injection pressure during injection was approximately 50% lower for all batches. The biggest difference in injection pressure occurred between batch 2 (1907 bar resp. 1304 bar) and batch 3 (1726 bar resp. 1111 bar) with a deviation of 10% for the casing geometry and 20% for the tension rod. Overall, it is noticeable that the relative changes in maximum injection pressure among the batches were consistent for both geometries. This means that process outliers and random correlations can be ruled out, which was also confirmed by the representative curve progressions during injection of the tension rods, as shown in Figure 19b. The different pressure levels were present throughout the injection phase and not just at the maximum injection pressure. The dip in pressure at the start of injection is related to the changes in flow path geometry as the cold runner system filled.

The resulting injection pressure was significantly influenced by the material viscosity and component geometry. The fluctuations in injection pressure therefore also support the measured differences in material viscosity (Figure 13 and Figure 14) and illustrate that a MFR deviation of under 3 g/min can lead to a geometry-dependent process fluctuation of up to 20%. Since a change in viscosity, without process adaptation, can be accompanied by a change in part weight, the fluctuations shown in the process data can influence the resulting part quality.

**Figure 20:**
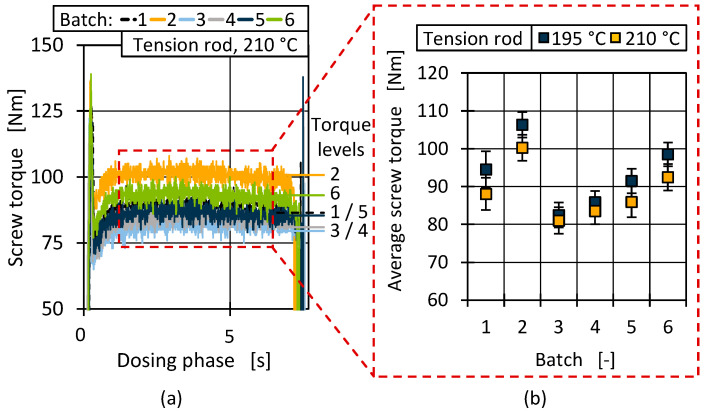
Representative torque levels during dosing phase (**a**) and average dosing torque for both processing temperatures (**b**).

To further analyse the plasticising and melt behaviour, the screw torque during dosing was evaluated. Due to acceleration and deceleration phenomena, the screw torque as a measure of material resistance during dosing was not analysed as a single (maximum) data point but as a curve [96]. Therefore, representative continuous screw data curves for the dosing phase are shown in Figure 20a. The curves show the previously described torque peaks where a distinction between batches is only possible to a limited extent. In comparison, clear differences in material behaviour can be observed in the steady-state dosing phase Figure 20a (red). Comparable to the injection pressure curves and the results of the rheological material evaluation, the necessary torque with the same dosing parameters varied among the batches.

The differences can be seen more clearly when determining the average screw torque in the constant dosing phase (Figure 20b). An increased temperature tended to reduce the viscosity and therefore also the necessary torque. However, a reproducible trend can be observed for both temperatures over the material batches, with batch 2 resulting in the highest (100 Nm resp. 106 Nm) and batch 3 in the lowest (about 81 Nm) torque. The fluctuation range and, thus, the stability of the dosing process was low and comparable for all batches. As stated before, the changes in viscosity mainly affected the processing conditions.

#### 3.3.1. Influence of Batch Fluctuations on Geometric and Mechanical Part Quality Stability

##### Part Weight

**Figure 21:**
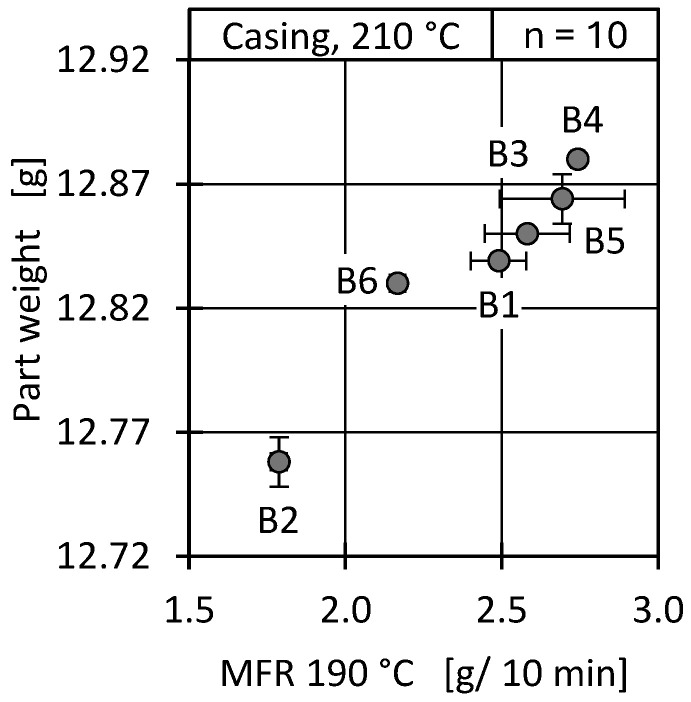
Part weight of the casing geometry over the measured MFR (190 °C) for all batches.

Component weight is a commonly used indicator of component quality consistency that is easy to measure and is often recorded inline on an industrial scale. Due to the pressure-controlled holding pressure phase, the material viscosity can influence the component filling and the resulting component weight [80]. With an increased viscosity (and, correspondingly, a decreasing MFR), the necessary pressure to fill the cavity increases. This has already been shown in the process data (Figure 19). Figure 21 shows that the increased pressure requirement is reflected in the part weight as well.

A lower batch MFR resulted in a lower part weight. The absolute weight variation between batches was marginal at a maximum of 13 mg, but industrially validated processes usually define a tolerance range, which may be narrow depending on the application.

##### Dimensional Accuracy

**Figure 22:**
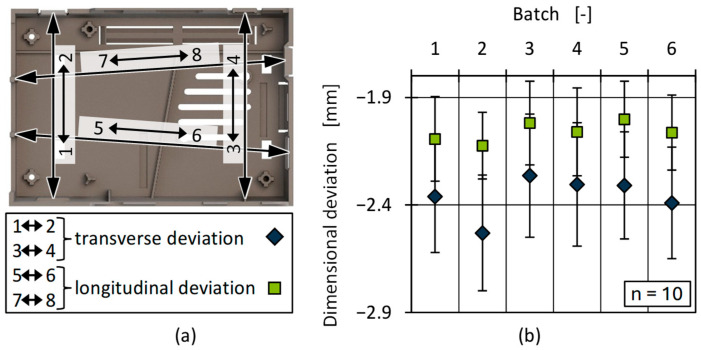
Positions of the measurement spans (**a**) and averaged transverse and longitudinal dimensional deviation for all batches (**b**).

As injection-moulded parts are often assembled into components, they are subject to tight dimensional tolerances. Differences in part dimensions cannot usually be represented by the part weight alone, even if a consistent part weight is regularly used as an indicator of its quality consistency. Under the same process conditions in the manufacturing process, the part dimensions of injection-moulded plastic parts are largely determined by the part warpage due to local shrinkage and geometry-dependent residual stresses during cooling [97]. A comparison of the variations in the part dimensions can be simplified by using defined measurements spans. Figure 22 shows the positions of those spans (a) as well as the mean values for the transverse and longitudinal dimensional deviations for all batches (b). The deviations were calculated by building the difference between the measured path lengths of the produced parts and the theoretical optimal part dimensions (see Section 2.3). The differences were averaged to generate a mean value for the transverse longitudinal dimensional deviation, respectively, which was compared among the batches.

Although the overall deviation of the batches was high in relation to the part dimensions, it was comparable to measurements conducted on virgin HDPE, given that HDPE generally has a high shrinkage potential. However, the results can be used to determine differences among the batches. All batches deviated by about 2 mm from the reference geometry. Due to the use of an optical measurement system rather than a tactile one, as well as averaging the values, the standard deviations were relatively high, and no statistically significant differences can be determined among the batches. Comparing only the averages, a slightly higher deviation occurred in batches 1, 2, and 6. For all batches, there was a tendency towards higher transverse than longitudinal deviation (overall average -2.36 mm vs. 2.06 mm). This could be related to the differences in rheological behaviour of the batches, which also led to differences in part weight. Further explanations could be derived from the relatively lower deviations of batches 3 und 5. Those batches showed the highest molecular weight and CH_3_/CH_2_ ratio (Figure 11 and Figure 12). The high CH_3_/CH_2_ ratio is an indicator for a higher PP content in batches 3 and 5. Since the presence of PP changes the crystallisation kinetics, nucleation effects can therefore also be a strong influencing factor regarding part shrinkage and the resulting dimensional accuracy [43].

##### Mechanical Properties

**Figure 23:**
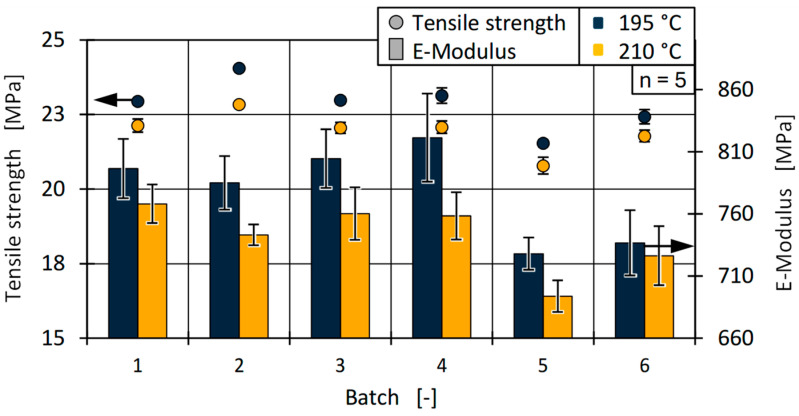
Tensile and strength and E-modulus of all batches for both cylinder temperatures.

In addition, the mechanical properties were analysed. For this purpose, both the maximum tensile strength and the modulus of elasticity were assessed. The results are shown in Figure 23 for all batches and both processing temperatures.

Regarding tensile strength, all batches showed similar tendencies at both temperatures, with a decrease in processing temperature increasing material strength. This can be attributed to an increased degree of crystallisation, possibly due to the reduced cooling rate during injection moulding or to potential degradation of the material at higher processing temperatures. It is notable that there was a relatively large difference in strength and stiffness between batches 4 and 5 (approximately 13% for the modulus of elasticity at 195 °C), which is discussed further below.

**Figure 24:**
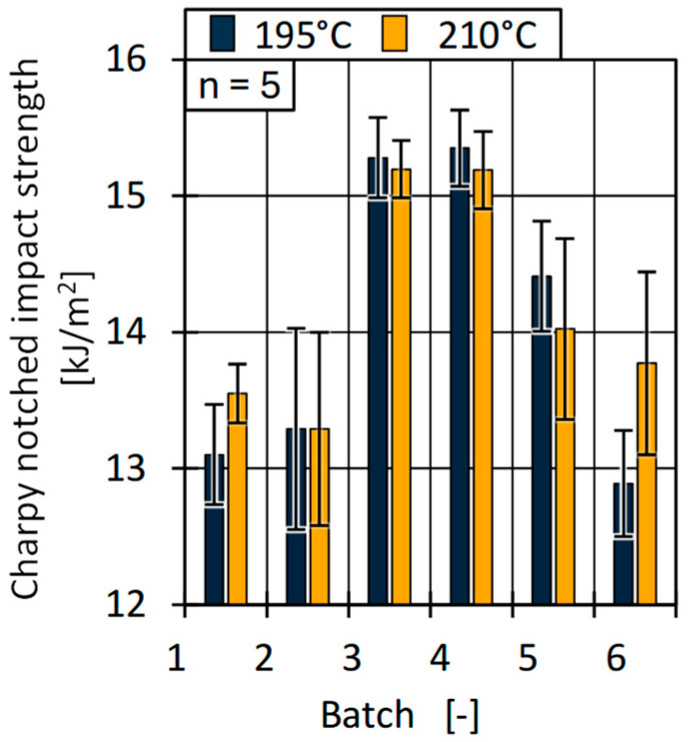
Determined notched Charpy impact strength for all batches.

In addition, the Charpy notched impact strength is shown in Figure 24. In contrast to the tensile strength and E-modulus, the impact strength reached a maximum of about 15 kJ/m^2^ for batches 3 and 4 and significantly lower values for batches 1, 2, and 6.

The results of the notched impact strength show a correlation with the rheological properties of the batches (MFR and HCR) as well as the process data (max. injection pressure) and the pvT behaviour. The trends suggest that a lower viscosity (higher MFR) is associated with a higher impact strength. Similar to the effect of part weight, a lower viscosity, at equal process settings, may lead to a more efficient holding pressure phase and, thus, a higher part density [98] and improved resistance to impact stress.

### 3.4. Determination of the Composition–Property Correlations

The presented results above comprehensively show the characteristics of the analysed PCR material batches and the resulting influences on processability and component properties. In the following, the results are summarised by determining Pearson’s correlations between the mean values of the individual characteristics. Even though the interpretation of the Pearson correlation depends on the scientific field, a (very) strong (positive or negative) linear correlation *r* can be expected if the correlation coefficient is greater than 0.7 resp. lower than −0.7 [99,100]. Non-linear correlations and interactions can also exist between the analysed data sets but were not evaluated further. Additionally, the influence of the deviations for the different data sets were not considered. If several values are available from the above presented results, only partial data was used to enable visualisation. The selected process and mechanical data refer to the analyses at a cylinder temperature of 210 °C and an MFR measurement at 230 °C. In case of dimensional deviations, only the transverse deviation is shown.

Table 3 shows the analysed parameters in relation to each other. Strong correlations ≥ |0.7| are highlighted and further discussed below. To determine whether the Pearson correlation coefficient is statistically significant, a *t*-test was carried out based on the t-distribution. A *p*-value of 0.05 was selected as the significance level. For the underlined values in the table, a *p*-value below 0.05 was reached and the correlation is considered statistically significant [101].

An overall observation is that many correlations can be attributed to the differences in viscosity between the batches. As a result, there are very high correlations, which correspond to the expectations of processability. As mentioned before, strong correlations (*r* ≥ |0.8|, partially *r* > |0.9|) exist between the **MFR** and the resulting process data (**p_t_**, **p_c_**, **t_t_**) but also the part weight, dimensions, and the impact strength. The correlations suggest that a relative increase in **MFR** leads to a decrease in injection pressure and torque but an increase in part weight, impact strength, and transversal dimensional deviation.

The more interesting correlations are those which could help explain the actual cause of the differences in viscosity. Due to further research and the presented results, an assumption can be made that the changes in viscosity can be (at least partially) explained by the deviations in foreign polymer (PP) content estimated through the PP melt enthalpy and crystallisation enthalpy (*r* > |0.7|). However, the significance test carried out did not show a statistically reliable linear correlation between viscosity and individual recyclate properties, such as the ash content or the PP content. Although it is known from the literature and practical experience that these components can influence viscosity, the result suggests that the relationship is likely non-linear or determined by complex interactions of several influencing variables, presumably also by additives. These could not be fully captured by the used statistical methods, in particular the linear correlation. To better identify non-linear or multivariate relationships, further analyses such as machine learning would be conceivable.

The differences in mechanical properties between the batches can be attributed to diverse effects. The molecular weight as well as the molecular weight distribution can influence strength and elasticity. Furthermore, the degree of crystallisation and co-crystallisation effects due to foreign polymers and additives correlate with the mechanical properties. For virgin HDPE, an increased molecular weight leads to increased tensile strength and E-modulus. For the analysed PCR batches, a different correlation can be determined, since the mechanical properties, tensile strength, and E-modulus (**σM**, **E_t_**), strongly negatively (partially significantly) correlated (−0.76, −0.93) with the molecular weight (**MW**) and the number of gels (−0.81, −0.81) determined during cast-film extrusion (**Gels**). This correlation is particularly evident in batch 5, which had a higher gel number and higher **MW** compared to the other batches. At the same time, the batch with the highest MW had the lowest tensile strength and the lowest E-modulus. In coherence with the formation of gels (Figure 17), it can be assumed that the increase in **MW** was a result of increased cross-linking due to the recycling and reprocessing of the material leading to defects and decreased matrix bonding and, thus, reducing the tensile strength overall. Consequently, a higher molecular weight in recycled materials does not necessarily lead to higher tensile properties, as is usually the case with virgin materials.

## 4. Conclusions and Outlook

This study investigated the influence of seasonal batch fluctuations on the material properties and processing conditions of post-consumer recycled HDPE. The results highlight the complexity of processing recycled materials, where seasonal changes and corresponding compositional variations have a significant impact on processing stability and part quality. In particular, foreign polymer interactions were found to be an important factor influencing viscosity, which, in turn, affects processing conditions as well as final part properties such as weight consistency or impact strength. Possible causes for the differences in viscosity were analysed and led to the assumptions that the influence of incompletely sorted-out lower-viscosity PP can be a reason for changes in rheological behaviour as well as residues from slip agents such as stearic acid, stearamide, or additives such as PEG.

However, viscosity alone does not sufficiently explain variations in mechanical properties or dimensional fluctuations. The molecular weight distribution determined by HT-GPC provides valuable insight into changes in tensile strength and E-modulus, but its interpretation differs from that of virgin HDPE. Higher molecular weight, which can result from increased cross-linking, leading to gel formation during film extrusion and defects in injection-moulded parts, can ultimately negatively affect mechanical performance. In this context, it is important to consider that machine manufactures and converters mainly focus on control and optimisation systems during extrusion and especially injection moulding, which are based on rheology and melt density. Since the rheological behaviour does not correlate with all the part characteristics, it will likely be increasingly difficult for converters to influence and stabilise properties such as tensile strength just through process optimisation over fluctuating PCR batches.

The research presented highlights the future importance of achieving a high and consistent quality of PCR materials to enable stable processes and constant part properties. Since recyclers have limited options for improving recyclate qualities (due to the given input waste stream), it is important to focus on “design for recycling” and reduced plastic type diversification already during the product development phase. This could help to mitigate the effects of seasonally changing consumer behaviour. With regard to packaging, intensive research will be required in the future to determine the extent to which the new packaging and packaging waste directive [1], for example, in the form of mono-material solutions, contributes to consistent, high-quality recycling streams and how these can be iteratively improved. In addition, strategies to mitigate processing instabilities—such as enhanced sorting technologies and material modification—should be explored to improve the reliability and performance of recycled HDPE in high-value applications. Overcoming these challenges will be essential to promote the sustainability and economic viability of the use of recycled polymers with year-round stable properties in industrial applications. Moreover, future research should focus on refining analytical methods to improve the characterisation of the compositional variations in PCR HDPE, explain their impact on processing and performance, and prioritise necessary analyses for converters. The integration of spectroscopic and rheological techniques could improve the understanding of molecular interactions and degradation mechanisms.

## Figures and Tables

**Table 1:** Injection moulding settings for both geometries.

	Geometry		
Setting	Tension Rod		Casing
Nozzle temperature [°C]	195	210	210
Mould temperature [°C]	40		
Injection volume rate [cm^3^/s]	50		
Injection time [s]	0.75		0.58
Holding pressure [bar]	550		800
Holding pressure time [s]	10		4
Screw rotation speed [mm/s]	200		
Back pressure [bar]	100		

**Table 2:** Melting and crystallisation temperatures for all batches determined via DSC.

Batch	T_M,PE_ [°C]	T_M,PP_ [°C]	T_C_ [°C]
1	132.2 ± 0.6	162.9 ± 0.7	116.9 ± 0.4
2	132.1 ± 0.0	162.9 ± 0.1	117.9 ± 0.3
3	132.3 ± 0.4	162.4 ± 0.3	117.7 ± 0.2
4	132.6 ± 0.7	162.4 ± 0.6	117.7 ± 0.5
5	131.8 ± 0.2	162.5 ± 0.7	117.6 ± 0.4
6	131.8 ± 0.1	162.7 ± 0.2	118.2 ± 0.3

**Table 3:** Pearson’s correlations for the determined material, process, and part characteristics.

	p_t_	p_c_	t_t_	m_c_	d_c_	MFR	E_t_	σ_M_	σ_I_	Ash	pvT	H_M, t_	H_M, PP_	H_C, t_	CH	MW	Gels
**p_t_**																	
**p_c_**	**0.99**																
**t_t_**	**0.82**	**0.84**															
**m_c_**	**−0.93**	**−0.97**	**−0.79**														
**d_c_**	**−0.95**	**−0.96**	−0.68	**0.96**													
**MFR**	**−0.96**	**−0.95**	**−0.81**	**0.85**	**0.91**												
**E_t_**	−0.22	−0.24	−0.33	0.11	0.04	0.38											
**σ_M_**	0.42	0.43	0.38	−0.53	−0.60	−0.25	**0.74**										
**σ_I_**	**−0.83**	**−0.83**	−0.44	**0.80**	**0.79**	**0.85**	0.28	−0.15									
**Ash**	−0.34	−0.38	**−0.73**	0.39	0.35	0.22	−0.01	−0.47	−0.20								
**pvT**	−0.59	−0.58	−0.33	0.56	**0.70**	0.69	0.06	−0.24	**0.76**	−0.32							
**H_M, t_**	**0.75**	**0.75**	**0.90**	**−0.70**	−0.67	−0.65	−0.20	0.43	−0.29	**−0.85**	0.01						
**H_M, PP_**	**−0.82**	**−0.84**	**−0.95**	**0.81**	**0.79**	**0.73**	0.15	−0.53	0.39	**0.82**	0.17	**−0.98**					
**H_C, t_**	**0.86**	**0.84**	0.61	**−0.82**	**−0.83**	**−0.72**	0.22	**0.70**	−0.64	−0.39	−0.34	**0.71**	**−0.75**				
**CH**	−0.64	−0.58	−0.45	0.51	**0.73**	0.66	−0.29	−0.61	0.53	0.03	**0.77**	−0.28	0.39	−0.64			
**MW**	−0.24	−0.22	−0.32	0.28	0.42	0.12	**−0.76**	**−0.93**	−0.09	0.47	0.18	−0.34	0.41	−0.51	0.65		
**Gels**	−0.05	−0.01	−0.11	0.04	0.15	−0.11	**−0.81**	**−0.81**	−0.30	0.47	−0.19	−0.31	0.29	−0.47	0.42	**0.90**	−0.05

Used abbreviations: **p_t_**: max. injection pressure (tension rod, 210 °C); **p_c_**: max. injection pressure (casing); **t_t_**: torque (tension rod, 210 °C); **m_c_**: part weight (casing); **d_c_**: dimension (casing, transverse); **MFR**: melt flow rate (230 °C, 5 kg); **E_t_**: E-modulus; **σ_M_**: tensile strength; **σ_I_**: impact strength; **Ash**: residual ash content; **pvT**: specific volume (200 °C, 800 bar); **H_M,t_**: total melt enthalpy; **H_M,PP_**: PP melt enthalpy; **H_C,t_**: total crystallisation enthalpy; **CH**: CH_3_/CH_2_ ratio; **MW**: molecular weight; **Gels**: total number of gels (>400 µm).

## Data Availability

Data set is available on request from the authors.

## Abbreviations

The following abbreviations are used in this manuscript:

BHT	Butylated hydroxytoluene
Đ	Dispersity
DSC	Differential scanning calorimetry
EVA	Ethylene vinyl acetate
FT-IR	Fourier-transform infrared spectroscopy
HCR	High-pressure capillary rheometry
HDPE	High-density polyethylene
HT-GPC	High-temperature gel permeation chromatography
LCB	Long-chain branching
MFR	Melt flow rate
MW	Molecular weight
OPC UA	Open Platform Communication Unified Architecture
PA	Polyamide
PCR	Post-consumer recycled
PEG	Polyethylene glycol
PET	Polyethylene terephthalate
PP	Polypropylene
pvT	Pressure- and temperature-dependent specific volume
SCB	Short-chain branching
T_C_	Crystallisation temperature
T_M_	Melting temperature
ΔH_C_	Crystallisation enthalpy
ΔH_M_	Melting enthalpy
